# Double reinsertion including Whitnall’s ligament in aponeurotic ptosis surgery: Comparison of results following simple aponeurosis reinsertion surgery and a combined reinsertion of the aponeurosis to Whitnall’s ligament for aponeurotic palpebral ptosis: a cohort study of 722 cases

**DOI:** 10.1007/s00417-020-05004-z

**Published:** 2021-01-11

**Authors:** Nuria Pfeiffer, Markus Pfeiffer, Alberto Reche Sainz, Nicolas Toledano Fernandez

**Affiliations:** 1 Lidclinic Pfeiffer, Garmischer Strasse 4, 86339 Munich, Germany; 2 University Hospital 12 Octubre, Madrid, Spain; 3 University Hospital Fuenlabrada, Madrid, Spain

**Keywords:** Ptosis, Whitnall’s ligament, Levator aponeurosis, Tarsus

## Abstract

**Purpose:**

The results of a technique with a double reinsertion of the aponeurosis to the tarsus and aponeurosis to Whitnall’s ligament (ATW) were compared with a simple reinsertion of the aponeurosis to the tarsus (AT) in acquired aponeurotic palpebral ptosis surgery.

**Methods:**

Analytical, observational, retrospective, cohort study. Seven hundred and twenty-two consecutive cases with acquired aponeurotic palpebral ptosis have been treated surgically between 2000 and 2012 and have been followed up for 5 years. The cases were divided into two cohorts according to the applied surgical technique (AT vs ATW).

**Results:**

The mean postoperative MRD after 1 month in cohort AT was 1 mm lower than in ATW (3 ± 0.9 mm vs 4 ± 1 mm). The mean MRD in the long-term follow-up (5 years) was 1 mm lower in cohort AT than in ATW (2.9 ± 1.5 mm vs 3.9 ± 0.9 mm). The rate of long-term recurrence (5 years) was 15% higher in A-T than in A-T-W (20% vs 5%). 70.5% of the eyes studied intra-surgically presented gaps between the Whitnall ligament and the aponeurosis, an anatomical area that we describe as the upper transition zone (UTZ). In an independent analysis, only those patients with open UTZ were evaluated and it was observed that those operated with A-T-W presented elevations greater than 1 mm compared to those operated with the AT technique (4 ± 0.9 mm A-T-W vs 2.8 ± 1 mm A-T) and a much lower recurrence rate (5.4% A-T vs 38.09% A-T-W).

**Conclusions:**

In our study, the A-T-W technique achieved better results in terms of palpebral elevation and fewer recurrences compared to the A-T technique in all cases studied with aponeurotic ptosis. However, it particularly demonstrates its superiority in patients with large gaps in the UTZ.

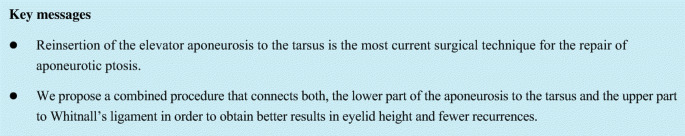

## Introduction

Palpebral ptosis is a frequent pathology but remains a challenge for the oculoplastic surgeon since it is not easy to achieve satisfactory results that last over time [1].

There are different types of palpebral ptosis and the acquired aponeurotic ptosis is the most frequent. This involutional type of palpebral ptosis is associated with age. The anatomical basis can be observed as a disconnection of the aponeurosis from the tarsal plate [2].

**Fig. 1:**
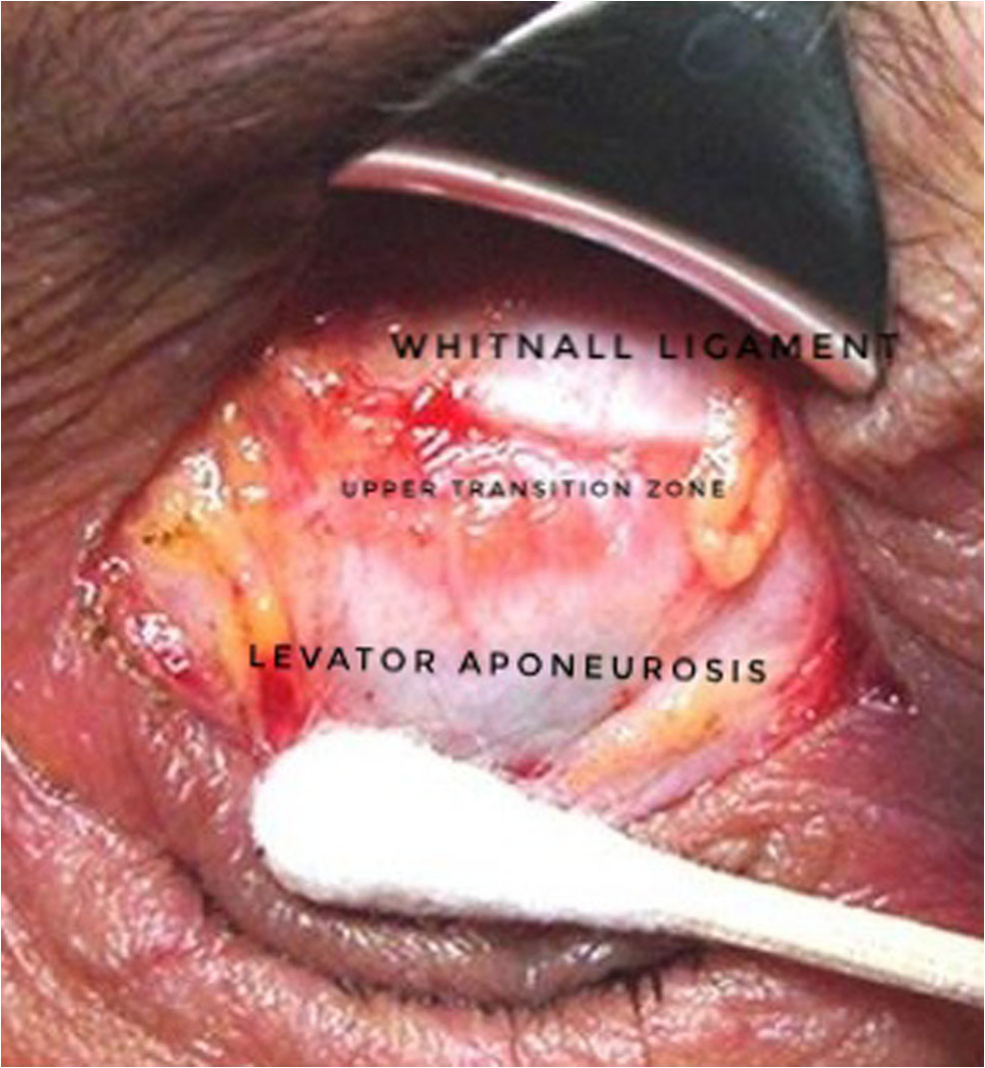
Intraoperative image showing an opened UTZ between the aponeurosis and the Whitnall ligament

Anatomically, the levator system of the upper eyelid is formed by three parallel arcs of fibrous tissue that are basic structures of the levator system: the tarsus, the aponeurosis of the elevator muscle, and Whitnall’s ligament [3]. Between Whitnall’s ligament and the upper aponeurosis, there is a transition with continuous or discontinuous fibrous tissue. For the purpose of this study, we name it the upper transition zone (UTZ). Under normal conditions Whitnall’s ligament is connected by fibrous tissue towards the aponeurosis leaving a central gap of 1–3 mm (UTZ). However, in certain cases, there may be a disinsertion of fibers or a fatty degeneration of the UTZ, producing larger separations between Whitnall’s ligament and the aponeurosis (Fig. 1) [4, 5].

There are multiple surgical techniques described for the treatment of this pathology. The most common surgery is the reinsertion of the aponeurosis to the tarsal plate (AT) [6].

## Justification

As ptosis surgery is related to less predictable results in comparison to other surgeries of eyelid pathology, we solely concentrate on improving the surgical technique [7]. Classical ptosis surgery like anterior levator resection, or posterior Muellerotomie includes shortening of the levator complex by resection and loss of elastic or muscular tissue. Alternative techniques avoid tissue loss and manipulate fibrous tissue ligaments to preserve their continuity. In this study, we propose an alternative surgical technique based on a double connection of the aponeurosis to the tarsus and to the Whitnall’s ligament (ATW) without any resection of tissue. Afterwards, we compare it with the simple connection of the aponeurosis to the tarsus (AT). The possible advantage is that the A-T-W technique not only repairs the disinsertion of the aponeurosis from the tarsus as the main cause of ptosis [8], but also corrects the disinsertion of the aponeurosis to Whitnall’s ligament, which can be another important factor of ptosis [9].

The aim of this study is to evaluate the proposed surgery ATW concerning the achievement of better palpebral elevations in the short and long term, but also with fewer recurrences than the classical A-T surgery.

## Material and methods

This is an analytical, observational, retrospective, cohort study. This study has been accepted by an ethical committee of clinical research. We analyzed 722 consecutive cases with acquired aponeurotic palpebral ptosis surgically intervened between 2000 and 2012. The null hypothesis (H_0_) of this work is the non-superiority or inferiority of the ATW technique compared to AT. The alternative hypothesis (H_1_) is the superiority of the ATW technique over AT. Since two surgical techniques are evaluated, to calculate the sample size, following the previous literature, an improvement of the 80% after surgical treatment in each group is estimated. To achieve an accuracy of 5% in estimating the ratio, with asymptotic confidence intervals normal to 95% bilateral, the minimum size was defined in 60 patients in each group. The inclusion criteria were patients with acquired palpebral ptosis with MRD < 2.5 mm, complete medical history, photographs (before and after surgery), operated by the same surgeon, and a minimum follow-up of 5 years. All patients who did not meet the previously named criteria were excluded. The patients were divided into two cohorts according to the technique (AT vs ATW) they were operated with. Patients were distributed in each group randomized according to the date of surgery, on even days AT was performed, on odd days ATW. The patients were evaluated preoperatively, intraoperatively, and postoperatively. Anatomical measurements of visual acuity, eyelid height (MRD), eyelid sulcus height and eyebrow height, function of the levator muscle, lid lag effect, and bell phenomenon were taken in all examinations. Intraoperatively, the macroscopic state of the upper transition zone was analyzed in all patients. The main postoperative success variables were margin reflex distance (MRD between 2.5 and 5.5 mm) in short (1 month) and long term (5 years), as well as the rate of recurrency. There were no preoperative and intraoperative statistical differences between both cohorts. Those postoperative MRD measurements higher than 5.5 mm were considered hypercorrections and those lower than 2.5 mm hypocorrections. All measurements were made by the same ophthalmologist.

**Fig. 2:**
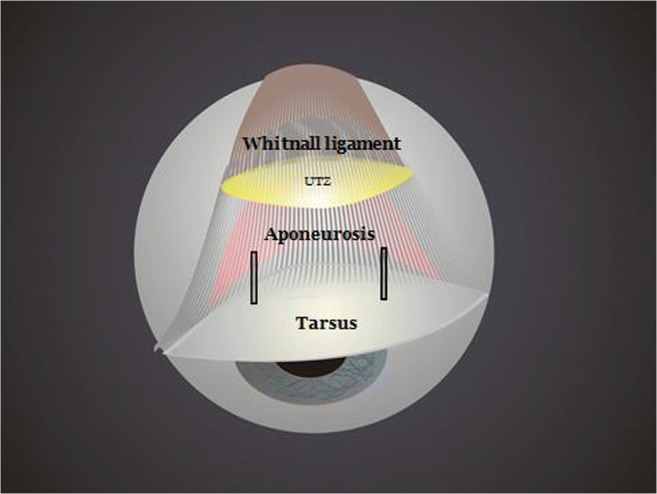
A-T technique. Simple reinsertion of the aponeurosis to the tarsus

**Fig. 3:**
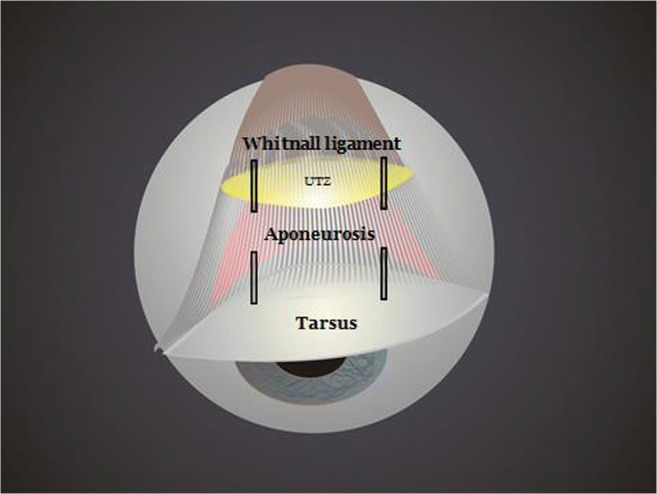
A-T technique. Simple reinsertion of the aponeurosis to the tarsus through double 6/0 double nylon sutures

**Fig. 4:**
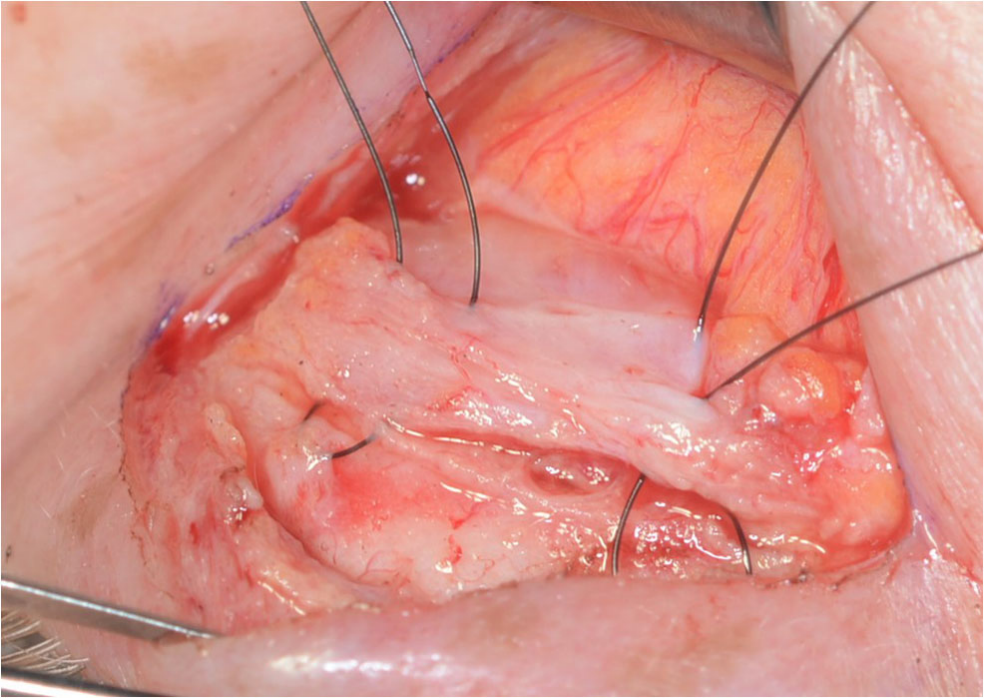
A-T technique. Sutures closed between tarsus and levator aponeurosis

**Fig. 5:**
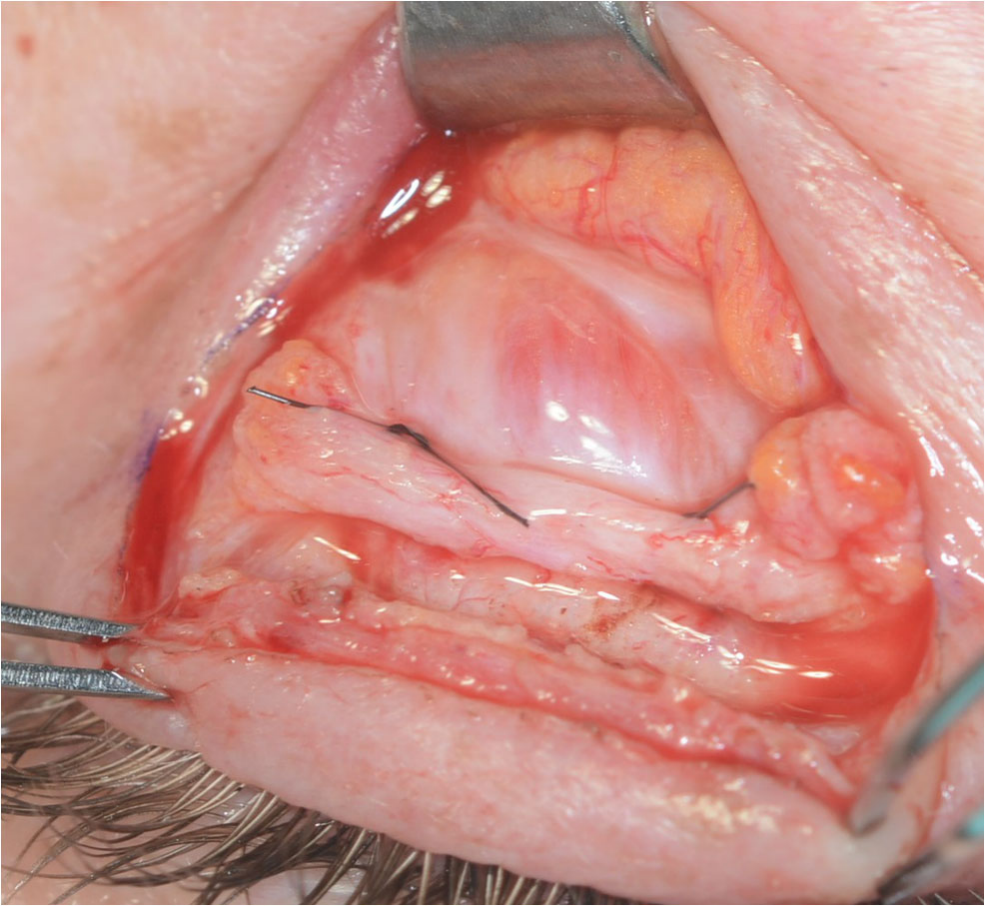
A-T-W technique. Double reinsertion of the aponeurosis to the tarsus and to the Whitnall ligament

**Fig. 6:**
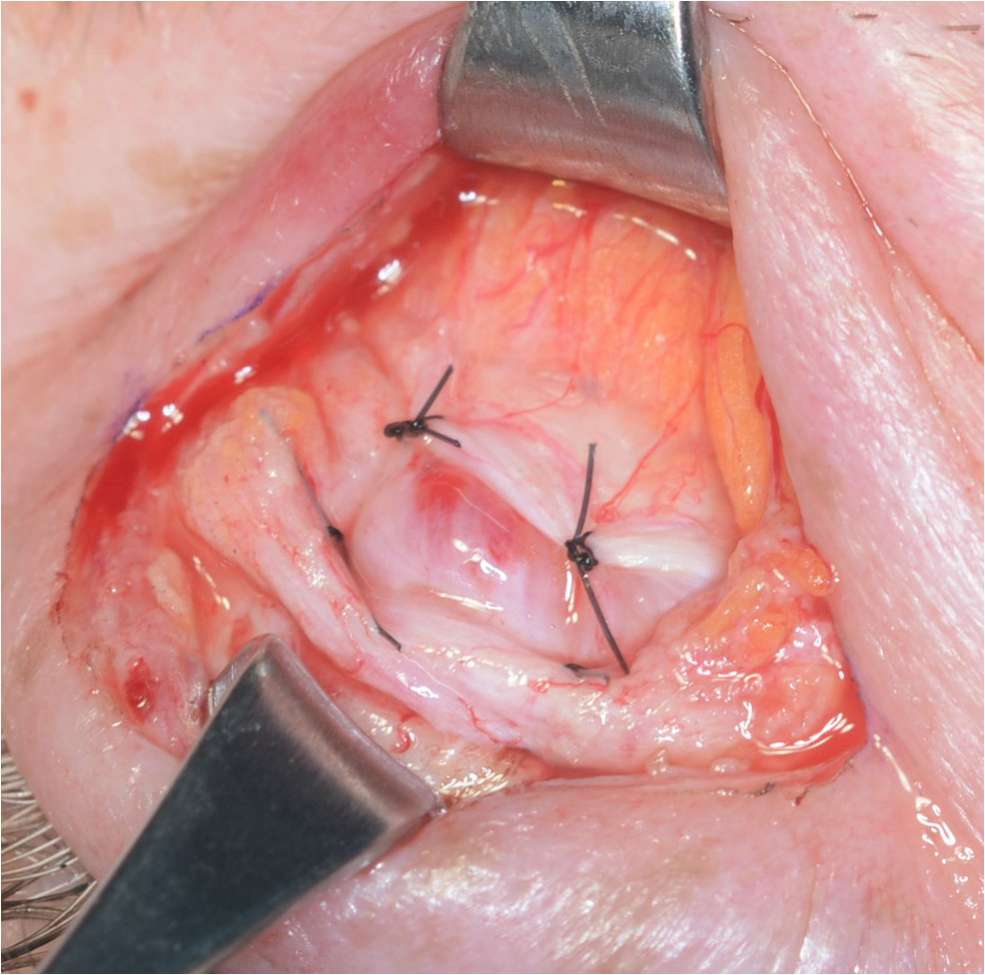
A-T-W technique. Double reinsertion of the aponeurosis to the tarsus and to de Whitnall ligament with 4 sutures

The anterior approach through the skin crease was performed on all patients. The level of the elevator system was reached by advancing through the skin, orbicular muscle, septum, and orbital fat by hydrodissection with 0.75 bupivacaine without adrenalin. In all cases, 6/0 nylon sutures were used. In the AT cohort, only the aponeurosis was sutured to the tarsus at two strategic points joining the central third with the lateral and medial thirds to achieve an arched shape of the eyelid and avoid vaulted forms (Figs. 2, 3, and 4). In the ATW cohort, the same sutures were set from the aponeurosis to the tarsus, but two sutures were also associated in parallel points, joining the aponeurosis to the Whitnall’s ligament (Figs. 5 and 6). All surgeries were performed by the same oculoplastic surgeon to avoid variation of surgical skills.

## Results

In our sample, the presurgical mean MRD in cohort AT was 0.8 mm (d.e. ± 1.1 mm) and in cohort ATW 0.7 mm (d.e. ± 1.1 mm). The mean postoperative MRD per month in cohort AT was 3 mm (d.e. ± 0.97 mm) and in cohort ATW 4 mm (d.e. ± 1.03 mm). This difference has a strong statistical significance (*p* < 0.01) and is clinically relevant. In the AT cohort, hypocorrections (MRD lower than 2.5 mm after surgery) were more frequent than in the cohort ATW (15.5% versus 7.1%). This difference is statistically significant (*p* < 0.01) (Table 1). Hypercorrections (MRD higher than 5.5 mm after surgery) were more frequent in cohort ATW than in A-T but the difference was not statistically significant.

**Table 1:** Principal results comparing both cohorts

	A-T	A-T-W
Pre surgical mean MRD	0.8 mm	0.7 mm
Mean MRD 1 month after surgery	3 mm	4 mm
Mean MRD 5 years after surgery	2.59 mm	3.78 mm
Rate of recurrence	20%	5%
Rate of hypocorrections	7.1%	15.5%

**Fig. 7:**
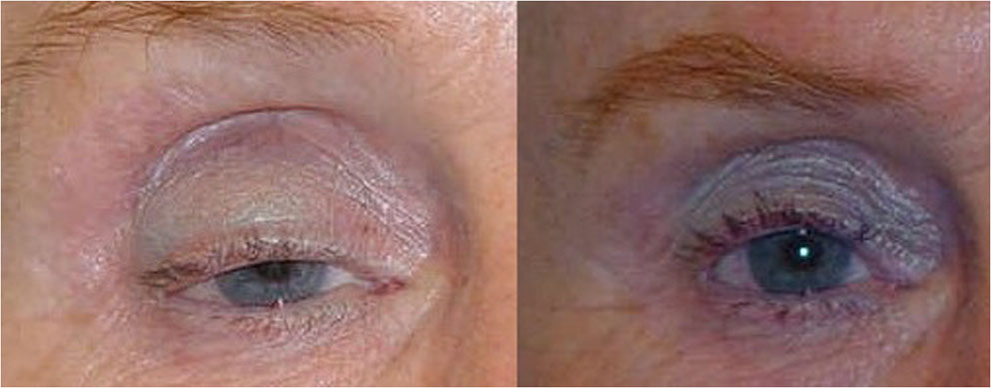
Results after operating with the A-T technique. 3 mm MRD

**Fig. 8:**
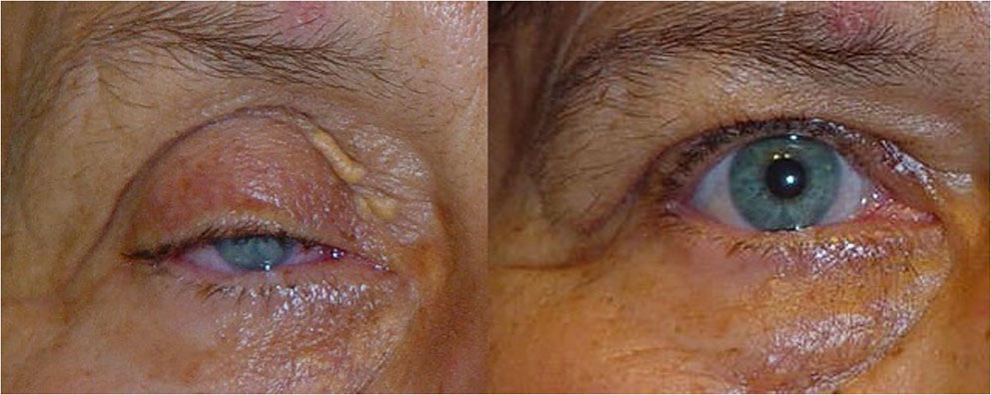
Results after operating with the A-T-W technique. 4.5 mm MRD

The long-term follow-up (5 years) of palpebral height is a fundamental variable to assess the effectiveness of surgery over time. The mean MRD in the long-term follow-up was 1 mm lower in A-T vs ATW : 2.59 mm (± 1.56 mm) vs 3.78 mm (± 0.92 mm) (*p* < 0.01) (Table 1) (Figs. 7 and 8).

The highest rate of recurrence in the AT cohort (20%) was statistically demonstrated (*p* < 0.01) with respect to ATW (5%) (Table 1). In the regression analysis, the most significant explanatory variable was the surgical technique. Consequently, the ATW technique appears as the determining factor. Using the ATW technique multiplies by 0.154 (or reduces by 82%) the possibility of recurrence.

The 70.5% of the eyes studied intra-surgically presented gaps (> 3 mm) of the UTZ. In an independent analysis, not randomized, studying only those patients with UTZ gaps, the mean MRD a month after surgery in those patients was greater if they were operated with the ATW technique (4 mm, of ± 0.975 mm) than if they were operated with AT (2.89 mm of ± 1.084 mm); in terms of recurrence with ATW, this was 5.4%, whereas with AT it was 38.09%, all this differences are statistically significant (*p* < 0.01) (Table 2).

**Table 2:** Results comparing both techniques in an independent analysis, not randomized, studying only those patients with UTZ gaps

	A-T	A-T-W
Mean MRD 1 month after surgery	2.89 mm	4
Recurrence	38.09%	5.4%

## Discussion

Multiple techniques have been described over the years to treat palpebral aponeurotic ptosis (Fasannela Servat, Blaskowitz, elevator resection, Müllerectomy) [1] but the most usual to date is the reinsertion of the aponeurosis to the tarsus as it fixes the main cause of ptosis, the disinsertion of the aponeurosis to the tarsus [7]. Suturing Whitnall’s ligament to the aponeurosis in congenital ptosis has been poorly described in the literature [9, 10], but a combined reinsertion of the levator aponeurosis to the tarsus and to the Whitnall ligament has not been evaluated in a study. Whitnall’s ligament has been proven to have almost the same motility as the levator aponeurosis [11]; therefore, we propose a technique that reinserts the aponeurosis to these two fundamental anchorage points (Whitnall’s ligament and tarsus).

Although our study is difficult to reproduce and would have greater validity, if carried out prospectively and randomized (simple, by blocks, stratified, or by minimization), we demonstrate a surgical alternative to correct palpebral ptosis, not described in literature, that tries to repair the elevator system without resections and maintain the entire elastic tissue of the aponeurosis and Whitnall’s [9].

The results may show the superiority of the ATW technique. We try to extent the theory on the pathophysiology of palpebral ptosis; although new studies that validate this idea are necessary, it is possible that the levator system is disintegrated at various anchorage points that can be repaired in surgery.

In our study, the ATW technique achieves better results in terms of palpebral elevation in the short (1 month) and long term (5 years) as well as fewer recurrences in all patients offering an overall anatomical restoration of the upper eyelid system [12], whereas the AT technique offers a partial restoration that leaves the UTZ open, facilitating recurrence. In an independent analysis studying only those patients with UTZ openings, the ATW technique offers very superior results with respect to AT. The status of the UTZ should be inspected during eyelid ptosis surgery, performing an ATW technique versus a simple AT could improve the results. In conclusion, in our work the alternative hypothesis (H_1_) rejects the null hypothesis (H_0_).

## Conclusions

In our study, the ATW technique achieves better results in terms of palpebral elevation and fewer recurrences compared to the classic reinsertion (AT technique). Especially in patients with gaps of the upper transition zone, it has shown to be more effective.

In our study, gaps in the UTZ are associated with acquired aponeurotic palpebral ptosis related to ptosis with a higher risk of recurrence.

According to our results, we can think that there are coalescing disinsertions of the aponeurosis apart from the main one of the tarsus, which may explain our results, although more studies are needed to validate this pathophysiological theory.
